# Genetic Diversity and Population Structure of *Sitodiplosis mosellana* in Northern China

**DOI:** 10.1371/journal.pone.0078415

**Published:** 2013-11-12

**Authors:** Yun Duan, Yu-qing Wu, Li-zhi Luo, Jin Miao, Zhong-jun Gong, Yue-li Jiang, Tong Li

**Affiliations:** 1 Institute of Plant Protection, Henan Academy of Agricultural Sciences, Key Laboratory of Crop Pest Control of Henan Province, Key Laboratory of Crop Integrated Pest Management of the Southern of North China, Ministry of Agriculture of the People's Republic of China, Zhengzhou, China; 2 Institute of Plant Protection, Chinese Academy of Agricultural Sciences, State Key Laboratory for Biology of Plant Diseases and Insect Pests, Beijing, China; Instituto de Higiene e Medicina Tropical, Portugal

## Abstract

The wheat midge, *Sitodiplosis mosellana*, is an important pest in Northern China. We tested the hypothesis that the population structure of this species arises during a range expansion over the past 30 years. This study used microsatellite and mitochondrial loci to conduct population genetic analysis of *S. mosellana* across its distribution range in China. We found strong genetic structure among the 16 studied populations, including two genetically distinct groups (the eastern and western groups), broadly consistent with the geography and habitat fragmentation. These results underline the importance of natural barriers in impeding dispersal and gene flow of *S. mosellana* populations. Low to moderate genetic diversity among the populations and moderate genetic differentiation (*F*_ST_ = 0.117) between the two groups were also found. The populations in the western group had lower genetic diversity, higher genetic differentiation and lower gene flow (*F*_ST_ = 0.116, *Nm* = 1.89) than those in the eastern group (*F*_ST_ = 0.049, *Nm* = 4.91). Genetic distance between populations was positively and significantly correlated with geographic distance (*r* = 0.56, *P*<0.001). The population history of this species provided no evidence for population expansion or bottlenecks in any of these populations. Our data suggest that the distribution of genetic diversity, genetic differentiation and population structure of *S. mosellana* have resulted from a historical event, reflecting its adaptation to diverse habitats and forming two different gene pools. These results may be the outcome of a combination of restricted gene flow due to geographical and environmental factors, population history, random processes of genetic drift and individual dispersal patterns. Given the current risk status of this species in China, this study can offer useful information for forecasting outbreaks and designing effective pest management programs.

## Introduction

The wheat midge (Géhin) *Sitodiplosis mosellana* (Diptera: Cecidomyiidae) is one of the most destructive pests of wheat, and is distributed in most wheat-producing regions of the world, including Europe, Asia, and North America [1]–[3]. *S. mosellana* was first detected in China in the early 1310s. This species has one generation per year. Females live for 3–7 days and lay 60–80 eggs. The larvae hatch after 4–7 days, crawl into the floret and feed on the surface of developing wheat kernels for 2–3 weeks. Once moist conditions are detected, the mature larvae drop to the ground, burrow into the soil and overwinter as diapausing cocooned larvae. Most overwintering larvae may remain in diapause for 1–2 years, though a few can maintain diapause for up to12 years [4]. Wheat midge infestation can result in kernels shriveling, cracking and deformity, and finally reduce crop yields and lower the grade of harvested grain [5]. In China, wheat midge outbreaks have occurred many times in the past 60 years, causing economic damage to wheat yields, especially in northern China [3].

Northern China is the major wheat-producing area in China, and is a region with complex topography (including plateaus, plains, rivers, lakes, basins, foothills and mountains) and variable climate. This region is also the main distribution area of *S. mosellana*. The geographical distribution of *S. mosellana* over the past 30 years has shifted to the northeast of about 400 km [6]. Continuing significant changes are expected during the coming decades. Since the beginning of the 21st century, *S. mosellana* outbreaks have continued to affect certain wheat fields [6]. For example, in 2005–2007, Henan and Hebei, which are the main wheat-growing areas in China, experienced serious damage with the loss of more than 2 million hectares of wheat. Its widespread agricultural impact and rapidly expanding range mean that although *S. mosellana* has been effectively controlled, it still remains a serious pest in some regions of China [3], [7].

Genetic diversity and population structure are important aspects of the population genetics of agricultural insects. Comparative studies of these aspects can therefore help to elucidate the factors affecting their population levels. The results of previous studies have shown that the genetic diversity and population structure of a species may be affected by several factors, such as climate change, environmental and ecological factors, natural barriers, human activities, migration and gene flow, and that these factors often act in combination [3], [8]–[11].

Molecular genetic techniques, such as simple sequence repeat and mtDNA analyses, can be used to conduct a genetic analysis of *S. mosellana*. The results of such an analysis will provide essential information for understanding possible local adaptation and dispersal patterns, and for further clarifying the relationship between genetic variation and outbreaks of wheat midge in China. However, the limited number of markers available for analyzing geographic populations and individuals means that few population genetic studies of *S. mosellana* have been conducted [5], [12]–[15]. Previous studies showed genetic variation and genetic differentiation among geographical populations of this species, and some degree of population structure. However, these studies covered a relatively small geographic area (not including populations from new outbreak areas) and a much smaller population (only 2–10 individuals), and used a limited number of molecular markers and limited statistical analyses [5], [12]–[15]. These results provided insufficient information for a clear understanding of the genetic diversity and population structure of this species.

Based on background knowledge, we used two molecular markers (microsatellites and mtDNA) that have been widely used in many insect population genetic studies [16]–[19] to detect the genetic diversity, genetic differentiation and population structure of *S. mosellana* across a broad geographic area in northern China. This is the first study to investigate the population genetics of *S. mosellana* comprehensively, and the results may provide more useful data for forecasting outbreaks and managing this species in China (e.g. by breeding resistant wheat varieties). These findings may also have significant implications for other regions where this species occurs.

## Materials and Methods

### Ethics statement

*Sitodiplosis mosellana* is a pest insect of wheat. The study of this pest is welcomed by farmers because understanding the behavior of this pest may be helpful to protect their wheat from pest damage. Thus, no specific permits were required for the described field studies.This study was carried out on private land, and we had got permission from the owners of these sites to conduct this study. Additionally, the field studies did not involve endangered or protected species.

### Sample collection and preparation

Soil samples containing larvae of *S. mosellana* were collected from 16 geographic sites in northern China from May to Sep. in 2009–2011 (Table 1), ranging from the east coast, the North China Plain to the Northwest Plateau (102°–118°E; 32°–40°N; spanning 16° longitude and 8° latitude and including an altitude gradient). Soil samples from five sites in a field, representing one geographical population of *S. mosellana*, were collected (each site, 10×10×20 cm) and then mixed together. Larvae (still in the soil) were maintained at 22±1°C, 70–75% relative humidity and a photoperiod of 14-h light∶10-h dark through pupation and adult emergence stages. Adults from each site were collected daily and stored individually in 75% ethanol at −20°C until DNA extraction was performed according to Collins et al. [20].

**Table 1:** Locations of 16 populations of *S. mosellana* collected in China.

Collection site	Code	Location	Altitude (m)
1.Shandong-Linyi	LY	118°20′E/35°02′N	65
2.Shandong-Jining	JN	116°35′E/35°24′N	59
3.Anhui-Funan	FN	115°35′E/32°37′N	32
4.Hebei-Xingtai	XT	114°30′E/37°03′N	69
5.Hebei-Xushui	XS	115°43′E/39°02′N	14
6.Tianjing	TJ	117°18′E/39°48′N	7
7.Beijing	BJ	116°37′E/40°17′N	59
8.Henan-Nanyang	NY	112°58′E/33°03′N	125
9.Henan-Huixian	HX	113°45′E/35°32′N	129
10.Henan-Luanchuan	LC	111°31′E/36°05′N	829
11.Shannxi-Huaxian	HuaX	109°46′E/34°30′N	369
12.Shannxi-Zhouzhi	ZZ	108°22′E/34°18′N	1158
13.Shanxi-Linfen	LF	111°31′E/36°05′N	457
14.Gansu-Lintao	LT	103°88′E/35°39′N	1886
15.Gansu-Wuwei	WW	102°61′E/37°94′N	1846
16.Ningxia-Yinchuan	YC	106°27′E/38°47′N	1102

Sites are labeled according to abbreviated location names for *S. mosellana*.

### Microsatellite analysis

All individuals were genotyped at four microsatellite loci: EW353, EW548, EW892 and Tig99 [21]. Forward primers were labeled with fluorescent dye (Sangon Biotech Co., Ltd., Shanghai, China). The primers used were: EW353-F: FAM-GAGTGGCAGGAAACAAGAGC, EW353 -R: TCATTGATGGAAGACTCACTT; EW548-F: ROX-TGCGAATCGATACCATAGGC, EW548-R: ATTGCCTGGAATTTTCCCTC; EW892-F: ROX-CCTCTCATTGATGCGTGTCT, EW892R: TTGAGTACCAGATTGCATTTCA; Tig99-F: FAM-GTGGAAATACGGTGCCATTT, Tig99-R: TCTTTCTCTCTCTCTGGCGG, respectively. The PCR reactions were carried out in a 20-µL reaction mixture containing 20–40 ng total DNA, 2.0 µL of 10× Taq DNA polymerase reaction buffer (Mg^2+^ free), 1.8 µL of 25 mmol/L Mg^2+^, 2 µL of 0.5 mmol/L dNTPs, 1.0 µL of 10 pmol/µL each primer, 11 µL ddH_2_O, 0.2 µL of Taq E (5 U/µL) (Takara, Shiga, Japan) and 2 µL of DNA template. Reactions were carried out using a SensoQuest LabCycler 2.2 (SensoQuest GmbH, Göttingen, Germany) thermocycler for 35 cycles. After initial denaturation for 3 min at 95°C, each cycle comprised denaturation at 94°C for 1 min, 45 s at the annealing temperature of each primer, 50 s extension at 72°C, with a final extension at 72°C for 10 min. Annealing temperatures varied from 55°C for EW353, 56°C for EW548 and Tig99 to 57°C for EW892. Following amplification, the products were visualized at Sangon Biotech Co., Ltd. (Shanghai, China) using an ABI 3730XL automated sequencer (Applied Biosystems, Foster City, CA, USA). Microsatellite alleles were analyzed using GeneMapper4.0 software (Applied Biosystems). Microsatellite data were checked for errors and null alleles with MICROCHECKER V2.2.3 [22].

### Mitochondrial DNA amplification and sequencing

Polymerase chain reaction (PCR) was used to amplify the target sequences from *ND4* and *COX3* mtDNA using the following primers developed in this study: ND4-F (5′- GAAATAGGAG-AAGATATATT-3′), ND4-R (5′-TTGAAATAAGATTAATTCCTAC-3′); COX3-F (5′-ACGA-GATGTAACTCGAGAAAG-3′), COX3-R (5′-ATCAAGCTGCAGCTTCAAATC-3′). Annealing temperatures were 45°C for *ND4* and 52°C for *COX3*. Following amplification, the products were sequenced by Sangon Biotech Co., Ltd. using the ABI-PRISM 3730 genetic analyzer (Applied Biosystems) using the same primers. The obtained sequence chromatograms were read with Chromas 2.33 (Technelysium Pty. Ltd., South Brisbane, Australia). All haplotypes have been submitted to GenBank (Accession numbers: KF030063–KF030131).

### Data analysis

Midges collected from each sampling site were assumed to represent local populations for the purposes of statistical analyses. To investigate the hypothesis that ecological, geographic and climatic factors may affect genetic variation, gene flow and population structure in this species, samples were divided into two groups throughout the region: the eastern group, including 10 populations (LY, JN, FN, XT, XS, TJ, BJ, NY, HX and LC); and the western group including six populations (HuaX, ZZ, LF, LT, WW and YC).

### Genetic diversity

For microsatellites, linkage disequilibrium was tested among all pairs of loci across all populations using GenePop 4.0 [23] and exact probability tests. An exact test for Hardy-Weinberg equilibrium (HWE) was conducted per locus and over all loci in each population using the same program. Corrections for multiple tests were performed by Bonferroni corrections. The extents of differences within and among populations were evaluated by basic statistical analyses including effective number of alleles (*N*_e_), observed heterozygosity (*H*_o_) and expected heterozygosity (*H*_e_) for each population, calculated using PopGen 32 (version 1.31) [24]. The number of alleles, allelic richness and gene diversity were calculated using FSTAT version 2.9.3.2 [25]. Correlation analyses of genetic diversity characteristics (allelic richness, expected heterozygosity and gene diversity) and their associations with latitude, longitude and altitude were conducted to test for possible clinal relationships.

Sequence data for mtDNA were assembled and aligned using Clustalx2 (version 2.0.12) [26] and MEGA 5 [27] and verified visually. DnaSP version 4.0 [28] was used to determine the number of variable sites, identify haplotypes, and calculate genetic diversity (haplotype diversity (*H_D_*) and nucleotide diversity (π) as defined by Nei [29].

### Genetic differentiation and gene flow among populations

For microsatellites, *F*_ST_ and *Nm* based on allele frequencies were calculated to estimate the degree of genetic differentiation over all populations using PopGen32. Pairwise *F*_ST_ values were used to estimate and assess the magnitude of differentiation among geographic populations using Arlequin v 3.5.1.2 [30] and tested for significant differences using 10,000 permutations. Pairwise *D_est_* values [31] for each population were also calculated using the web based resource SMOGD v1.2.5 [32]. We used 1,000 bootstrap replicates and the harmonic mean of *D_est_* across loci. For mtDNA *ND4*, pairwise *F*_ST_ values based on haplotype frequencies were calculated using the program Arlequin v 3.5.1.2.

Haplotype diversity (*H_D_*), intrapopulation diversity (*H_S_*), total genetic diversity (*H_T_*), population differentiation (*G*_ST_) and gene flow (*Nm*) were also compared in DnaSP between the eastern and western groups of *S. mosellana*.

To determine if genetic and geographic distances between populations were significantly correlated, isolation by distance (IBD) was examined by testing the correlation between pairwise *F*_ST_ and geographical distance using the Mantel test [33] (using GENEPOP 4.0 for microsatellites). IBD was also tested between the eastern and western populations. The linear distances between sampling sites were estimated using Google Earth (http://earth.google.com) with their coordinates. For testing of statistical significance, 10,000 permutations in Mantel tests were used to test the null hypothesis that genetic distance and geographical distance were independent. These results were plotted in SPSS 12.0 for Windows (SPSS Inc., Chicago, IL, USA).

### Population genetic structure

The Bayesian approach implemented in STRUCTURE version 2.2.3 [34] was used to analyze the geographic structures of *S. mosellana* populations using multilocus genotype data. This program uses a coalescent genetic approach to cluster similar multilocus genotypes into *K* clusters, regardless of an individual's geographical origin. We conducted eight independent runs for each value of *K* ranging from 1–12 using the admixture model and correlated allele frequencies. To detect the true *K* present in microsatellite data, an ad-hoc measure Δ*K* [34] from *K*1–*K*12 was used across all runs. Each run consisted of a burn-in of 10,000 steps, followed by 20,000 Markov chain Monte Carlo repetitions.

To apportion variance between the eastern and western groups of 16 *S. mosellana* populations identified by pairwise *F*_ST_ and STRUCTURE, hierarchical analysis of molecular variance (AMOVA) [35] based on *F*_ST_ (using mtDNA *ND4* haplotype frequencies) was carried out using Arlequin. The AMOVA program allows the hierarchical partitioning of the variance components into three components: within populations, among populations within groups and among groups. Significance of fixation indices was also tested using a nonparametric permutation approach with 2,000 permutations using the same software.

### Individual assignment and migrant detection

Each individual was assigned to a group (*K* = 2), previously inferred by STRUCTURE software using the Bayesian approach. Individuals were assigned to a group if the proportion ancestry ≥0.7; when no group was ≥0.7, the individual was unassigned and considered to be of mixed ancestry. Population assignment tests were also performed across all sites in this software (*K* = 5). Due to generally high levels of admixture, a threshold value of 70% was used when assigning membership of the sampling sites in the determined groups.

Assignment of individuals to their most likely population of origin was also performed with GENECLASS2 software [36]. This software was also used to identify *S. mosellana* first-generation migrant individuals using a partially Bayesian method [37] and Monte Carlo resampling algorithm [38] with 10,000 simulated individuals and type I error of 0.01. The probability that a certain individual came from a particular population was calculated using the *L*_h_/*L*_max_ likelihood test statistic to determine the critical value of *L*_h_/*L*_max_ beyond which individuals were assumed to be migrants.

### Phylogenetic relationships among populations and haplotypes

In order to explore the hierarchical relationships among *S. mosellana* populations further, POPTREE 2 software [39] was used to construct an unrooted neighbor-joining (NJ) tree based on values of Nei's genetic distance [40] calculated in the same software by 1,000 bootstraps. MEGA 5 was used to construct an unrooted NJ tree based on values of genetic distance between *ND4* haplotypes by 1,000 bootstraps.

Compared with conventional phylogenetic trees, haplotype networks can enhance the relationships among haplotypes and are preferable for intraspecific analyses. Unrooted networks of haplotype of *ND4* and *COX3* were therefore constructed in NETWORK 4.6.1.0 [41] by a median-joining method. We defined five groups of *S. mosellana* populations according to the results of STRUCTURE (*K* = 5) (group A: LY, JN, FN, XT, XS, TJ, BJ, NY and HX; group B: LC; group C: HuaX and ZZ; group D: LF and LT; group E: WW and YC).

### Population demographic history and neutrality test

Demographic history changes were analyzed for *S. mosellana* using two neutrality tests (Tajima's *D* [42] and Fu's *F_S_* [43]) across 16 geographic populations and pairwise mismatch distributions for all populations combined and two groups separately using mtDNA. We calculated neutrality-test statistics in these populations with 1,000 permutations in Arlequin and DnaSP. Neutrality tests were used as an indication of recent population expansion when the null hypothesis of neutrality was rejected due to significant negative values (*P*<0.05 for *D* and *F_S_*). According to coalescent theory, a population usually exhibits a unimodal mismatch distribution following a recent population demographic or range expansion [44].

The program BOTTLENECK v1.2.02 [45] was used to look for genetic signatures of a recent bottleneck in each population. The Wilcoxon's signed rank test [46] using three mutation models (e.g. the infinite allele model (IAM), the strict stepwise mutation model (SMM), and the two-phase model (TPM)) recommended by Piry et al. [47] was applied with 10,000 replications. A qualitative descriptor of the allele frequency distribution (mode-shift indicator) which discriminates between bottlenecked and stable populations was also used.

## Results

The four microsatellites were amplified in 374 individuals. Considerable variation was observed at all microsatellite loci. All four loci investigated were polymorphic both within and among populations. There was no evidence for linkage disequilibrium within any of the four loci (*P*>0.002 for all after Bonferroni corrections). Most populations deviated significantly from HWE (*P*<0.01) (excluding LY, FN, XT and LC). Microchecker confirmed the presence of null alleles in three out of four loci (EW353, EW548 and EW892). Single locus deviations from HWE (after Bonferroni corrections with *P*<0.001) were attributed to heterozygote deficiency in 21 out of 64 tests. However, these loci did not show consistent deviations across all populations. We therefore assumed that processes causing this nonequilibrium were specific to those populations, and we thus continued to include those loci in subsequent analyses.

For mtDNA, the sequence alignment length of 16 populations of *S. mosellana* was reduced to 603 for *ND4* and 472 for *COX3* to ensure no missing data across 320 samples. No insertions or deletions were detected in any of the sequences. In total, 42 haplotypes for *ND4* and 27 haplotypes for *COX3* were identified in these populations. Populations in the eastern and western groups exhibited haplotype polymorphism for each of the two genes.

### Population genetic diversity

Basic summary statistics of genetic diversity are presented in Table 2. Analysis showed that these populations of *S. mosellana* contained a substantial fraction of genetic variation. The mean allele number (A) values across microsatellite loci ranged from 3.50 in WW to 7.00 in HuaX, with a mean value of 5.75. Observed heterozygosity (*H*_o_) values and expected heterozygosity (*H*_e_) values across loci ranged from 0.32–0.79 and 0.45–0.72, respectively. Excluding four populations of ZZ, LF, WW and YC, observed heterozygosity (*H*_o_) values were lower than expected heterozygosity (*H*_e_) values. Allelic richness (*Ar*) per population ranged from 3.09–6.64. The mean gene diversity across loci was 0.64 with the lowest in WW (0.44) and the highest in ZZ (0.72), indicating a high level of information of the chosen microsatellite. The inbreeding coefficient (*Fis*) ranged from 0.02–0.61. Combining *ND4* and *COX3*, 15 out of 16 populations exhibited comparatively high haplotype diversity (excluding WW). The haplotype diversity and nucleotide diversity of these populations ranged from 0.28±0.016 to 0.82±0.025 and 0.0013±0.0005 to 0.0058±0.0004, respectively. Reduced levels of mtDNA genetic diversity were observed in three populations of LT, WW and YC compared with the other 13 populations.

**Table 2:** Genetic diversity based on four microsatellite loci (using PopGen 32) from 374 samples and mtDNA sequences (*ND4* and *COX3*) (using FSTAT v2.9.3.2) from 320 samples of *S. mosellana*.

Code	Microsatellite loci								mtDNA (*ND4*+*COX3*)		
	N	A	Ne	Ho	He	Ar	GD	Fis	N	Hd	π
1.LY	24	5.25	3.07	0.52	0.62	4.69	0.62	0.23	21	0.76(±0.018)	0.0044(±0.0004)
2.JN	24	6.75	4.57	0.32	0.71	6.03	0.65	0.33	21	0.72(±0.021)	0.0039(±0.0003)
3.FN	24	7.50	4.68	0.56	0.69	6.64	0.70	0.04	21	0.54(±0.023)	0.0037(±0.0002)
4.XT	24	6.00	3.81	0.45	0.71	5.50	0.71	0.22	23	0.67(±0.014)	0.0045(±0.0005)
5.XS	24	6.50	3.72	0.60	0.71	6.06	0.71	0.46	21	0.72(±0.017)	0.0045(±0.0003)
6.TJ	24	6.25	3.99	0.47	0.67	5.61	0.67	0.20	24	0.64(±0.025)	0.0038(±0.0004)
7.BJ	24	5.50	3.61	0.55	0.62	5.10	0.64	0.30	23	0.66(±0.011)	0.0039(±0.0002)
8.NY	24	6.75	4.30	0.58	0.66	5.96	0.65	0.40	20	0.68(±0.013)	0.0038(±0.0005)
9.HX	24	6.25	4.08	0.52	0.71	5.81	0.70	0.32	20	0.69(±0.019)	0.0040(±0.0004)
10.LC	24	6.00	3.35	0.40	0.62	5.36	0.64	0.02	22	0.82(±0.025)	0.0055(±0.0003)
11.HuaX	24	7.00	3.86	0.54	0.69	6.31	0.68	0.33	20	0.66(±0.023)	0.0038(±0.0002)
12.ZZ	24	5.75	3.99	0.72	0.72	5.23	0.72	0.61	21	0.53(±0.009)	0.0058(±0.0004)
13.LF	24	4.25	2.20	0.79	0.53	3.92	0.53	0.60	23	0.68(±0.018)	0.0045(±0.0004)
14.LT	14	5.00	2.58	0.55	0.61	5.00	0.61	0.27	14	0.61(±0.015)	0.0013(±0.0005)
15.WW	24	3.50	1.86	0.75	0.45	3.09	0.44	0.46	20	0.28(±0.016)	0.0018(±0.0002)
16.YC	24	3.75	2.43	0.65	0.58	3.58	0.58	0.39	23	0.64(±0.021)	0.0027(±0.0003)

N: sample size; A: mean allele number; Ne: effective number of alleles; Ho: observed heterozygosity; He: expected heterozygosity; Ar: allele richness; GD: mean Nei's genetic diversity; Fis: inbreeding coefficient; Hd: haplotype diversity; π: nucleotide diversity.

The present microsatellite data indicated that many populations (12 out of 16 populations) had a deficiency of heterozygotes with significantly positive F values (*P*<0.001). More generally, the eastern populations showed higher genetic diversity (mean allele number: 6.28, mean effective number of alleles: 3.92, mean alleles richness: 5.68) compared with the western populations (mean allele number: 4.88, mean effective number of alleles: 2.82, mean alleles richness: 4.52). This phenomenon was supported by mtDNA analysis.

Analysis of correlations between geographic variables and diversity parameters indicated that allelic richness, expected heterozygosity and genetic diversity were all negatively correlated with altitude (*r* = −0.629, *P*<0.01; *r* = −0.601, *P*<0.05; *r* = −0.588, *P*<0.05) and positively correlated with longitude (*r* = 0.569, *P*<0.05; *r* = 0.562, *P*<0.05; *r* = 0.553, *P*<0.05). Altitudinal trends were usually stronger than longitudinal ones, especially in terms of allelic richness measures. There were no remarkable correlations (*P*>0.05) between latitude and several diversity parameters in this study.

### Genetic differentiation and gene flow among populations

Genetic differentiation of all populations was measured using two different statistics (*F*_ST_ and *D_est_*) based on microsatellite data (Table 3). There was significant genetic differentiation among populations based on *F*_ST_ estimates. The results indicated that 10 populations in the eastern region were completely different from six populations in the western part. Pairwise *F*_ST_ values ranged from 0.006–0.265 (mean 0.095), and pairwise *D_est_* values ranged from 0.000–0.431 (mean 0.157). The pairwise *D_est_* values showed an overall similar pattern to the pairwise *F*_ST_ values, though the *D_est_* values were on average slightly higher. An overall randomization test of population differentiation was significant for all loci combined (*P*<0.001).

**Table 3:** Pairwise genetic distances across all populations of *S. mosellana* based on microsatellite data.

Code	1	2	3	4	5	6	7	8	9	10	11	12	13	14	15	16
1.LY	0.000	0.016	0.037	0.039	0.087	0.020	0.024	0.036	0.144	0.165	0.307	0.472	0.447	0.236	0.333	0.424
2.JN	0.012	0.000	−0.025	0.019	0.031	0.000	0.040	0.024	0.111	0.107	0.239	0.359	0.339	0.186	0.280	0.359
3.FN	0.022	0.010	0.000	0.002	0.027	0.000	0.028	0.039	0.150	0.097	0.229	0.329	0.337	0.171	0.301	0.34
4.XT	0.037	0.017	0.004	0.000	0.008	0.000	0.037	0.043	0.125	0.055	0.255	0.373	0.310	0.116	0.321	0.294
5.XS	0.057*	0.023	0.018	0.011	0.000	0.000	0.083	0.043	0.044	0.041	0.201	0.364	0.188	0.070	0.218	0.208
6.TJ	0.019	0.000	−0.006	0.000	0.002	0.000	0.006	0.010	0.078	0.021	0.210	0.357	0.276	0.131	0.268	0.334
7.BJ	0.027	0.021	0.022	0.018	0.039	0.013	0.000	0.028	0.095	0.105	0.278	0.431	0.325	0.163	0.246	0.318
8.NY	0.030	0.012	0.019	0.029	0.032	0.007	0.023	0.000	0.043	0.046	0.075	0.302	0.269	0.103	0.178	0.245
9.HX	0.084*	0.066*	0.061*	0.057*	0.043	0.052*	0.061*	0.031	0.000	0.032	0.106	0.234	0.114	0.061	0.113	0.141
10.LC	0.109**	0.070*	0.059*	0.051*	0.053*	0.043	0.069*	0.029	0.028	0.000	0.072	0.154	0.137	0.027	0.106	0.173
11.HuaX	0.160**	0.120**	0.107**	0.120**	0.107**	0.113**	0.139**	0.077*	0.061*	0.045	0.000	0.066	0.114	0.095	0.135	0.155
12.ZZ	0.205**	0.176**	0.146**	0.145**	0.136**	0.156**	0.174**	0.141**	0.108**	0.107**	0.048	0.000	0.256	0.225	0.296	0.311
13.LF	0.266**	0.199**	0.180**	0.183**	0.140**	0.176**	0.214**	0.168**	0.104**	0.091*	0.097*	0.143**	0.000	0.076	0.114	0.149
14.LT	0.170**	0.110**	0.103**	0.101**	0.056*	0.093**	0.116**	0.086*	0.059*	0.047	0.081*	0.129**	0.075*	0.000	0.025	0.021
15.WW	0.270**	0.213**	0.210**	0.232**	0.188**	0.211**	0.217**	0.170**	0.133**	0.140**	0.147**	0.223**	0.190**	0.064*	0.000	0.013
16.YC	0.250**	0.190**	0.177**	0.178**	0.139**	0.182**	0.193**	0.159**	0.115**	0.131**	0.124**	0.172**	0.150**	0.031	0.035	0.000

Pairwise *D_est_* (using SMOGD v1.2.5) estimates are presented above the diagonal, and *F*_ST_ (using Arlequin v3.5.1.2) estimates are presented below it.

Significance of comparisons indicated as follows:

* *P*<0.05;

** *P*<0.001.

Pairwise *F*_ST_ values among the eastern group ranged from 0.014–0.107, and among the western group from 0.021–0.267, suggesting significant genetic differentiation and low levels of gene flow (*F*_ST_ = 0.116, *Nm* = 1.891) among the western populations compared with the eastern populations (*F*_ST_ = 0.046, *Nm* = 4.907). The mean *F*_ST_ value between the eastern and western groups was 0.117. All the investigated loci contributed to this population differentiation (each individual locus *P*<0.001).

mtDNA analysis using Arlequin software showed similar results to the microsatellite data (Table S1) with *F*_ST_ values ranging from 0.22–0.45 and highly significant differentiation for exact tests across all populations, suggesting that the differentiation signal among all populations in mtDNA was stronger than that in microsatellites. DnaSP analysis indicated *G*_ST_ = 0.212, *Nm* = 0.39 for all populations; *G*_ST_ = 0.059, *Nm* = 3.96 for the eastern group; and *G*_ST_ = 0.078, *Nm* = 2.64 for the western group. Based on mtDNA *ND4* and *COX3*, the total haplotype diversity was 0.852, and the within-population diversity was 0.5065. *H*_ST_ of the total population was 0.237, indicating a strong differentiation among populations (*P*<0.001), among the eastern populations (*H_D_* = 0.767, *H_S_* = 0.693, *H*_ST_ = 0.099) and among the western populations (*H_D_* = 0.691, *H_S_* = 0.570, *H*_ST_ = 0.182). Although comparison of gene flow between the eastern and western groups was significant (*Nm*>1), there was insufficient evidence to prevent genetic differentiation.

**Figure 1:**
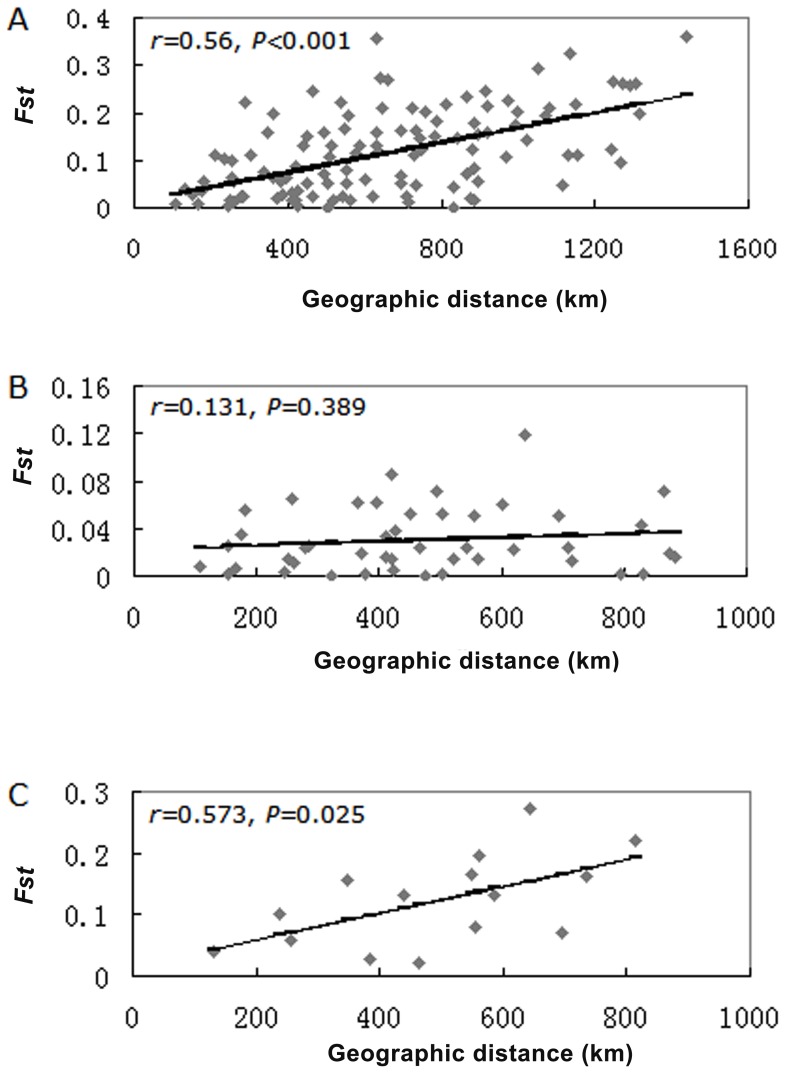
Relationships between pairwise *F*_ST_ values and geographical distances for *S. mosellana* populations. (A) 16 *S. mosellana* populations (*r* = 0.560, *P*<0.001). (B) 10 *S. mosellana* populations of the eastern group (*r* = 0.131, *P* = 0.389). (C) Six *S. mosellana* populations of the western group (*r* = 0.573, *P* = 0.025).

Applying the Mantel test, significant positive relationships between genetic and geographic distances were found over the 16 *S. mosellana* populations (*F*_ST_ vs km; *r* = 0.56 and *P*<0.001) (Figure 1A) and within the western group of six populations (Figure 1B) (*F*_ST_ vs km; *r* = 0.573 and *P* = 0.025), suggesting that the genetic population differentiation (gene flow) of this species may follow an IBD pattern. However, there was no evidence to indicate a correlation between genetic and geographic distances within the eastern group of 10 populations (*F*_ST_ vs km; *r* = 0.131 and *P* = 0.389) (Figure 1C).

### Population genetic structure

**Figure 2:**
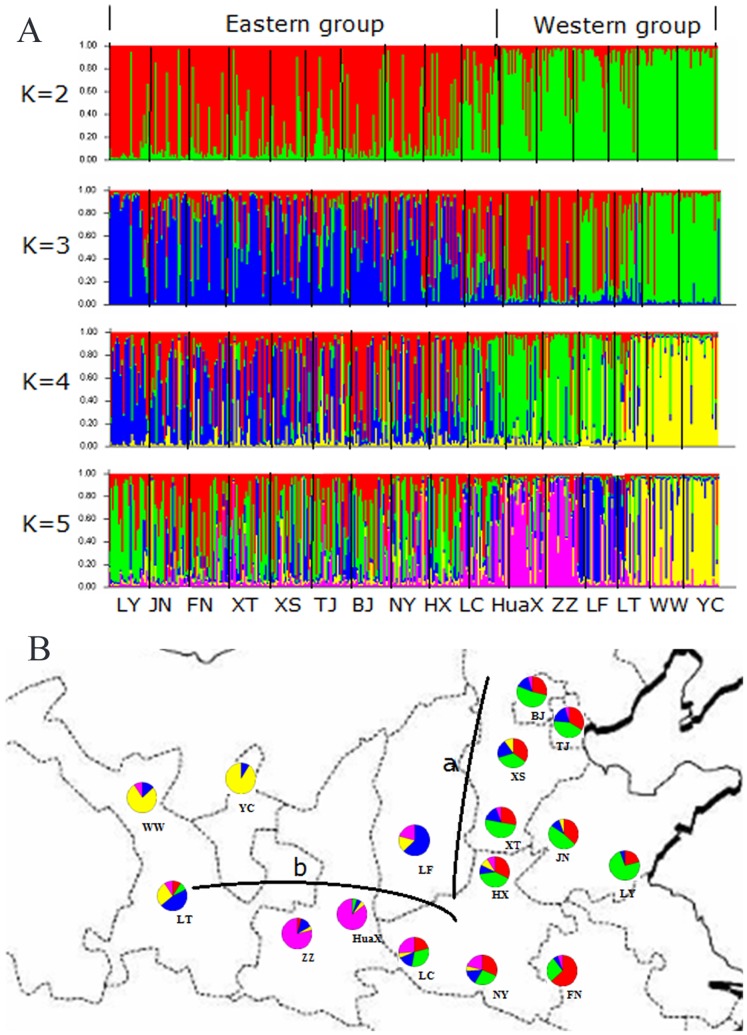
Population structure across 16 populations of *S. mosellana* obtained using STRUCTURE (for microsatellite data). (A) Individual Bayesian assignment probabilities for *K* = 2 to *K* = 5. Individuals are represented as thin vertical lines partitioned into segments corresponding to the inferred genetic clusters as indicated by color. (B) Schematic map of China showing sampling sites for *S. mosellana*. Pie charts show the mean membership fractions of each of the five genetic clusters in 16 populations. a: Taihang mountains; b: Qinling mountains.

To investigate the natural population structure and infer the relationships among *S. mosellana* populations, we used an admixture model implemented in STRUCTURE software to explore different numbers of populations *K* to the population structure based on microsatellite data. Considerable population structure was detected with LY+JN+FN+XS+XT+TJ+BJ+NY+HX+LC and HuaX+ZZ+LF+LT+WW+YC as two independent groups at *K* = 2, which was consistent with the hypothesis that these populations could be divided into two groups (the eastern and western groups), broadly corresponding to the two geographical areas (Figure 2A). Further structuring indicated divergence of HuaX and ZZ from the other western populations at *K* = 3. With *K* = 4, the eastern populations were apparently divided into two groups. The Bayesian clustering method detected significant genetic clusters among the western populations, with locations of HuaX and ZZ comprising one cluster, LF and LT comprising the second cluster, and WW and YC comprising the third cluster at *K* = 5. Optimal *K* estimated the most likely number of populations at *K* = 5 and most populations had a clear allocation to one of the five groups. A more fine-grained population structure was present at *K* = 5, and the subdivision still closely followed the geography (Figure 2B). The geographical structuring of mtDNA haplotypes among these populations was significant between the eastern and western groups.

Assuming that the genetic groups inferred from the Bayesian analysis represented two gene pools, we performed AMOVA for these populations. Similar results were also obtained when AMOVA was carried out considering the eastern and western groups based on mtDNA analysis, showing significant variation (50.14%; *φCT* = 0.501, *P*<0.001) among groups and within populations (43.73%; *φST* = 0.563, *P*<0.001), while variation among populations within groups was 6.13% (φ*SC* = 0.123, *P*<0.001) (Table S2). Overall, population differentiation using mtDNA was significant among the two groups, and was also significant when comparing the western and eastern populations (AMOVA, *P*<0.0001).

### Individual assignment and migrant detection

Individual assignment by STRUCTURE analysis indicated that 65–100% of the individuals from 15 populations (excluding LC) were assigned with membership probabilities of ≥70% (Table 4). A total of 327 out of 374 (87.4%) individuals were correctly assigned to a group (*K* = 2), with 176 individuals assigned to the eastern group and 123 individuals retained in the western group. Twenty-eight individuals were identified as migrants and 47 individuals as of mixed ancestry. Compared to the western populations (mean 81.3%), the eastern populations (mean 74.5%) tended to have lower assignment accuracy on average. The assignment test was successful for some populations (e.g. 100% assigned for WW and YC populations). Furthermore, with membership probabilities of ≥70%, the LC population was a mixture of insects from two groups, and may represent an intermediate type between the eastern and western populations.

**Table 4:** Assignment of individuals from 16 populations of *S. mosellana* to the eastern and western groups through Bayesian analysis (*K* = 2).

Code	N	East group	Western group	NA	%A
1.LY	24	21	2	1	88
2.JN	24	17	2	5	71
3.FN	24	20	2	2	84
4.XT	24	20	3	1	84
5.XS	24	17	3	4	71
6.TJ	24	18	1	5	75
7.BJ	24	20	2	2	83
8.NY	24	15	3	6	63
9.HX	24	18	4	2	75
10.LC	24	10	7	7	42
11.HuaX	24	2	18	4	75
12.ZZ	24	2	18	4	75
13.LF	24	1	22	1	92
14.LT	14	1	10	3	71
15.WW	24	0	24	0	100
16.YC	24	0	24	0	100

Each cell contains the number of individuals from each population assigned to the group with q≥0.70. NA: not assigned = number of individuals not assigned to any group; A%: percent assigned to a genetic group.

The detection of migrants using GENECLASS produced very similar results to that using STRUCTURE. Twenty-four individuals were identified as migrants (*P*<0.01), with 18 out of 134 (13.4%) from the western populations as present-generation migrants, while only six out of 216 (2.7%) from the eastern populations (excluding LC) were identified as migrants. No migrants originating from the WW and YC populations were detected in other populations.

### Phylogenetic relationships among populations and haplotypes

**Figure 3:**
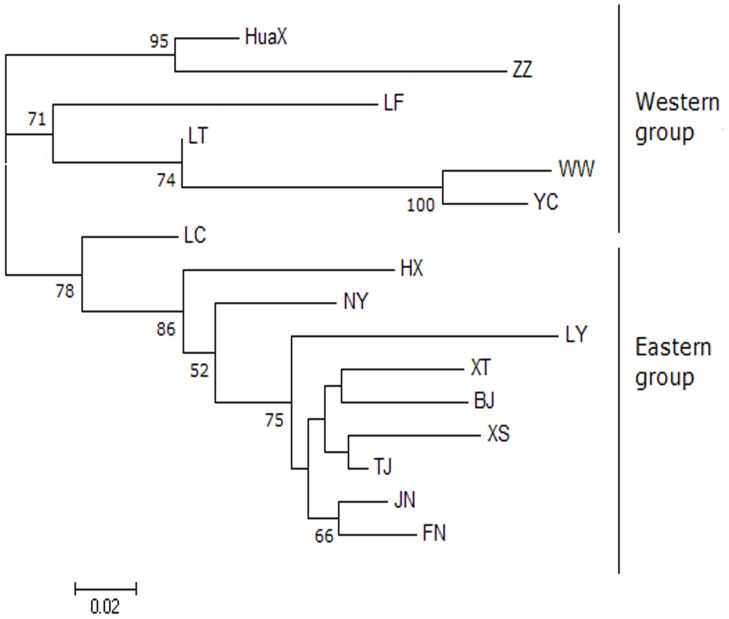
Neighbor-joining tree of 16 populations of *S. mosellana* based on Nei's genetic distances using allele frequencies of the four microsatellite loci. Bootstrap support above 50% (10,000 replicates) is indicated by gray branches.

The unrooted NJ dendrogram obtained from Nei's genetic distance (Figure 3) showed that 16 populations of *S. mosellana* were clearly segregated into two discrete groups (the eastern and western groups), which was consistent with the results analyzed by STRUCTURE. LC was separated from other nine populations in the eastern group with 78% bootstrap support, and significant clustering was observed among the western populations.

**Figure 4:**
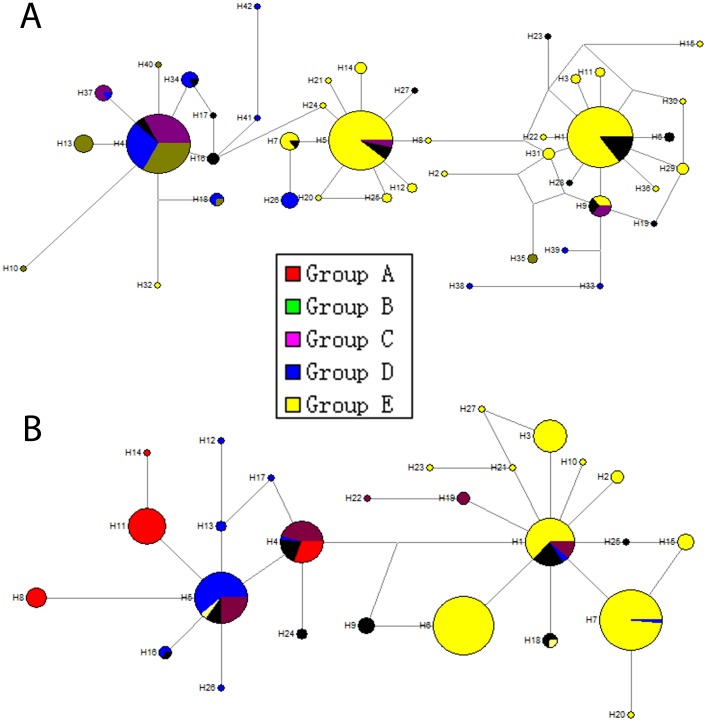
Median-joining network showing phylogeographic structure for the mtDNA *ND4* (A) and *COX3* (B) haplotypes of *S. mosellana*. Inferred median vectors are switched off for clarity. Each circle represents a haplotype, and circle size is proportional to haplotype frequency. Colors indicate the proportion of individuals sampled in different populations within the study area. Branch lengths are proportional to the number of S substitutions per nucleotide site. Group A: LY+JN+FN+XT+XS+TJ+BJ+NY+HX; Group B: LC; Group C: HuaX+ZZ; Group D: LF+LT; Group E: WW+YC.

The unrooted NJ tree of 42 haplotypes of *ND4* (Figure S1) and haplotype network analyses for *ND4* and *COX3* (*K* = 5) (Figure 4), showed that the haplotypes of the two genes were divided into two clades, while that from LC was divided into two groups. mtDNA relationships showed that haplotypes in the first clade were all from the eastern populations, while those in the second clade were divided into two branches; one branch from the eastern group and the other from the western group.

### Population demographic history and neutrality test

Neutrality tests were applied in grouped and non-grouped *S. mosellana* populations using mtDNA *ND4*. Tajima's *D* and Fu's *F_S_* tests were not significant (*P*>0.05) for all populations (Table S3) and the model of population expansion could be rejected, suggesting that these populations had not experienced rapid expansion. However, the model of population expansion could not be rejected using Fu's *F_S_* tests (*P*<0.05) for the eastern and western groups, and when all populations were combined. The mismatch distribution analysis of overall populations of *S. mosellana* tended to be unimodal (Figure S2). Separate analyses of the mismatch distribution of the eastern and western groups yielded similar results. These results indicated that this species had recently undergone a rapid expansion in China.

Bottleneck analysis indicated that no population of *S. mosellana* displayed significant heterozygosity excess (*P*>0.05) (Table S3) using Wilcoxon's signed rank tests with three mutation models of SMM, IAM and TPM, suggesting no genetic bottleneck in these populations caused by mutation-drift equilibrium. In addition, no shift in the frequency distribution of alleles and a normal L-shaped curve also indicated that the studied populations had not undergone bottleneck events in their recent history.

## Discussion

Recent advances in statistics and population genetics make it possible to investigate how geographical (e.g. landscape, latitude, longitude and altitude) and environmental (e.g. temperature and precipitation) factors affect the genetic diversity and population structure of a species [48], [49]. *S. mosellana* is widely distributed in Northern China, which includes many different geographic and biogeographic areas and can be divided into eastern and western regions according to geographic position. The results of the present study indicated that *S. mosellana* populations across its geographic distribution could also be divided into eastern and western groups in terms of their adaptations to different geographical and ecological environments in northern China. Significant differences in geographical and environmental factors between the two regions have led to great differences in genetic diversity, genetic differentiation and population structure of *S. mosellana*, which has been demonstrated at several scales.

### Genetic diversity

Genetic diversity is the basis of an organism's ability to adapt to changes in its environment, and can be affected by many factors [50]. Previous studies showed evidence of genetic variation among *S. mosellana* populations [5], [14]. However, they provided insufficient information regarding the genetic diversity of this species and the relationships between geographic variables and genetic diversity. The present study revealed that the studied populations of *S. mosellana* (excluding WW) harbor moderate to high genetic diversity, indicating their ability to adapt to varying environmental conditions. The eastern populations exhibit a higher degree of genetic diversity than the western populations, suggesting that fragmented habitats, migration (decreased or eliminated) between populations and genetic drift have probably contributed to the loss of diversity of the populations in the western region (especially in WW and YC). *S. mosellana* has recently undergone a substantial northeastern range expansion in China, including recent establishments in Tianjing and Beijing, which may be a cause of the low levels of genetic variation among the eastern populations. Analyses of the relationships between geographic variables (longitude and altitude) and genetic diversity (allelic richness, expected heterozygosity and gene diversity) showed that the former are important factors leading to differences in genetic diversity among *S. mosellana* populations.

### Genetic differentiation and gene flow among populations

Previous studies found obvious genetic differentiation between spring wheat-region and winter wheat-region groups among *S. mosellana* populations [5], [14]. The results of this study indicate that *S. mosellana* populations exhibit significant genetic differentiation, with *F*_ST_ values ranging from low to high among these populations. IBD tests identified a positive correlation between genetic and geographic distances in the populations, especially among the western group, suggesting that these populations are differentiated by a process of IBD, and that genetic drift is a much stronger force than gene flow. The present findings are partly consistent with those of prior studies [5], [14]. However, no IBD pattern was detected among the eastern populations, which may be due to region-wide expansion and stronger gene flow among these populations.

The *F*_ST_ (*D_est_*) values also revealed significant genetic differentiation between the eastern and western groups (*F*_ST_ = 0.11), indicating that they are in two different gene pools and have evolved independently for several hundreds of thousands of years. AMOVA analysis based on mtDNA data indicated high genetic differentiation among populations, with about 50% of the genetic diversity that existed among groups.

Compared to the eastern populations, higher genetic differentiation and lower gene flow were detected among the western populations, which may be the result of broken topography with numerous mountains and small plateaus characterized by very diverse climates in this area, leading to predominantly small-scale wheat growing. The populations of YC and WW had substantially higher levels of *F*_ST_, implying that drift may play a much larger role in determining allele frequencies in these populations than in other populations.

The geographical structuring of haplotypes was significantly different between the eastern and western groups, which could be divided into two groups. The results suggest that mountains (e.g. the Taihang mountains, which were formed during the mountain-building processes of the Jurassic period and have historically formed an obstacle to movement between Shanxi and Hebei) have been a major geographic barrier limiting gene flow between the eastern and western groups in this species, with significant isolation between the populations on either side of the Taihang mountains. The geographic barrier to gene flow in *S. mosellana* represented by the Taihang mountains may reflect this organism's dispersal ability and its adaptation to climatic variables. Mountains have also been shown to act as geographic barriers shaping the population structure of the other terrestrial species *Locusta migratoria* [51] and *Paeonia rockii* [52].

### Population structure

We tested the hypothesis that the population structure of *S. mosellana* arises during range shift in Northern China. A previous study found that *S. mosellana* could be divided into three groups: a spring wheat-region group, a winter wheat-region group and a mixed winter and spring wheat-region group [5]. Our results provide new insights into the population structure of this species and suggest that the populations can be divided into two groups: the eastern group and the western group. In this study, various analyses indicated that these populations exhibited significantly different population structures, reflecting the ancient polymorphisms of this species. The results also indicated that the range-wide phylogeographical structure of *S. mosellana* was mainly characterized by geographic isolation. The Bayesian clustering method revealed the presence of two distinct (eastern and western) lineages of *S. mosellana*, corresponding largely to patterns of habitat fragmentation and geographic origin.

The weak signal for division into five groups, combined with the high frequency of accurate assignments, suggests that fragmentation of the *S. mosellana* populations is not static. Compared with the eastern populations, the western *S. mosellana* populations are significantly differentiated from each other and more substructured. This study indicates that the complex and diverse topographic configuration in northern China (especially in the western region) might have played an important role in shaping the genetic divergence and population structure of *S. mosellana*. The mountains in particular have played a more significant role in the genetic substructuring of *S. mosellana* populations than linear distance, and the current results suggest that the Taihang and Qinling mountains may be considered as isolated management units in terms of control efforts.

### Detection of migrants and admixed individuals

Migration analyses detected a high number of potential migrants mainly among the western populations (excluding the populations of WW and YC), indicating asymmetric migration from the western populations to the eastern populations. However, this is apparently not sufficient to overwhelm the effect of drift in the isolated areas and to maintain genetic similarity across the western range of *S. mosellana*. Notably, the present study identified the presence of admixed individuals in most of these populations, suggesting that migration of *S. mosellana* occurred historically, and that this species is not only able to move between localities on occasion, but is also able to reproduce in the new area, thereby contributing to the genetic diversity of subpopulations.

This study indicates that the migration of *S. mosellana* may be affected by topography, wind, water and human activities. Our data also showed that the Qinling mountains could be considered as an important dispersal corridor allowing genetic exchange, as well as identifying isolated areas. The relatively strong global population structure and low degree of genetic variation within populations suggest that *S. mosellana* has a low capacity for active dispersal, but can disperse passively over long distances by wind, water and human-mediated transport [53].

## Conclusions

The widespread agricultural impact and rapid range expansions of *S. mosellana* make it necessary to understand its population structure and population dynamics in a mixed landscape in China. However, this aspect has been poorly studied to date. The results of the current genetic analyses of *S. mosellana* have important implications for the development of more effective and preventive pest management strategies in China, and for predicting population responses to climate change. To the best of our knowledge, this study is the most comprehensive report on the population genetics of *S. mosellana* at multiple scales across a broad geographic area in China. However, *S. mosellana* has a broad geographic distribution, and the diverse topography within its native range means that it shows strong regional subdivision in China. More detailed inferences about its phylogenetic and phylogeographic patterns are likely to be studied in the future, and require further sampling throughout China.

## Supporting Information

Figure S1 Neighbor-joining tree of haplotypes of *ND4* based on genetic distances. Bootstrap support above 50% (10,000 replicates) is indicated by gray branches.
(TIF)

Figure S2 **Mismatch distributions of pairwise nucleotide differences for total populations of** ***S. mosellana*** **using gene sequences of** ***ND4*** **(A) and** ***COX3*** **(B).** Solid lines show the observed frequency distributions and dashed lines show the distribution expected under the sudden-expansion model.
(TIF)

Table S1 **Pairwise genetic distances across all populations of** ***S. mosellana*** **based on mtDNA** ***ND4*** **using Arlequin v3.5.1.2.**
(DOC)

Table S2 **Analysis of molecular variance of** ***S. mosellana*** **based on mtDNA** ***ND4*** **(** ***K*** **= 2).**
(DOC)

Table S3 **Analyses of natural tests of Tajima's** ***D*** **and Fu's** ***F_s_*** **and bottlenecks in 16 populations of** ***S. mosellana*** **.**
(DOC)
