# Supplementary figures and images for: Genetic Diversity and Population Structure of Sitodiplosis mosellana in Northern China
Source: PLoS One. 2013 Nov 12;8(11):e78415. doi: 10.1371/journal.pone.0078415 (PMC3827046; doi:10.1371/journal.pone.0078415)

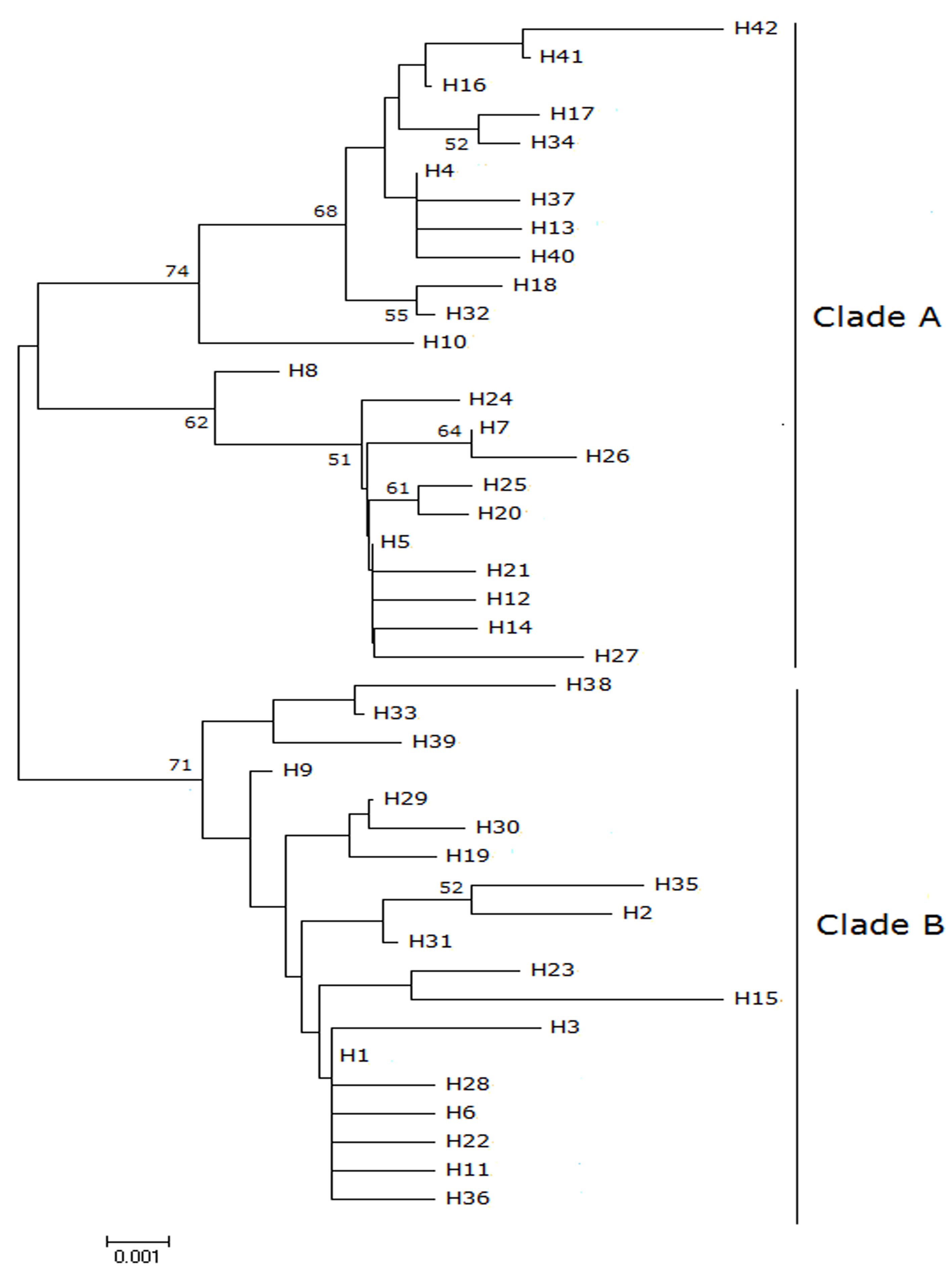

Supplement: Figure S1 — Neighbor-joining tree of haplotypes of ND4 based on genetic distances. Bootstrap support above 50% (10,000 replicates) is indicated by gray branches. (TIF) [file pone.0078415.s001.tif]

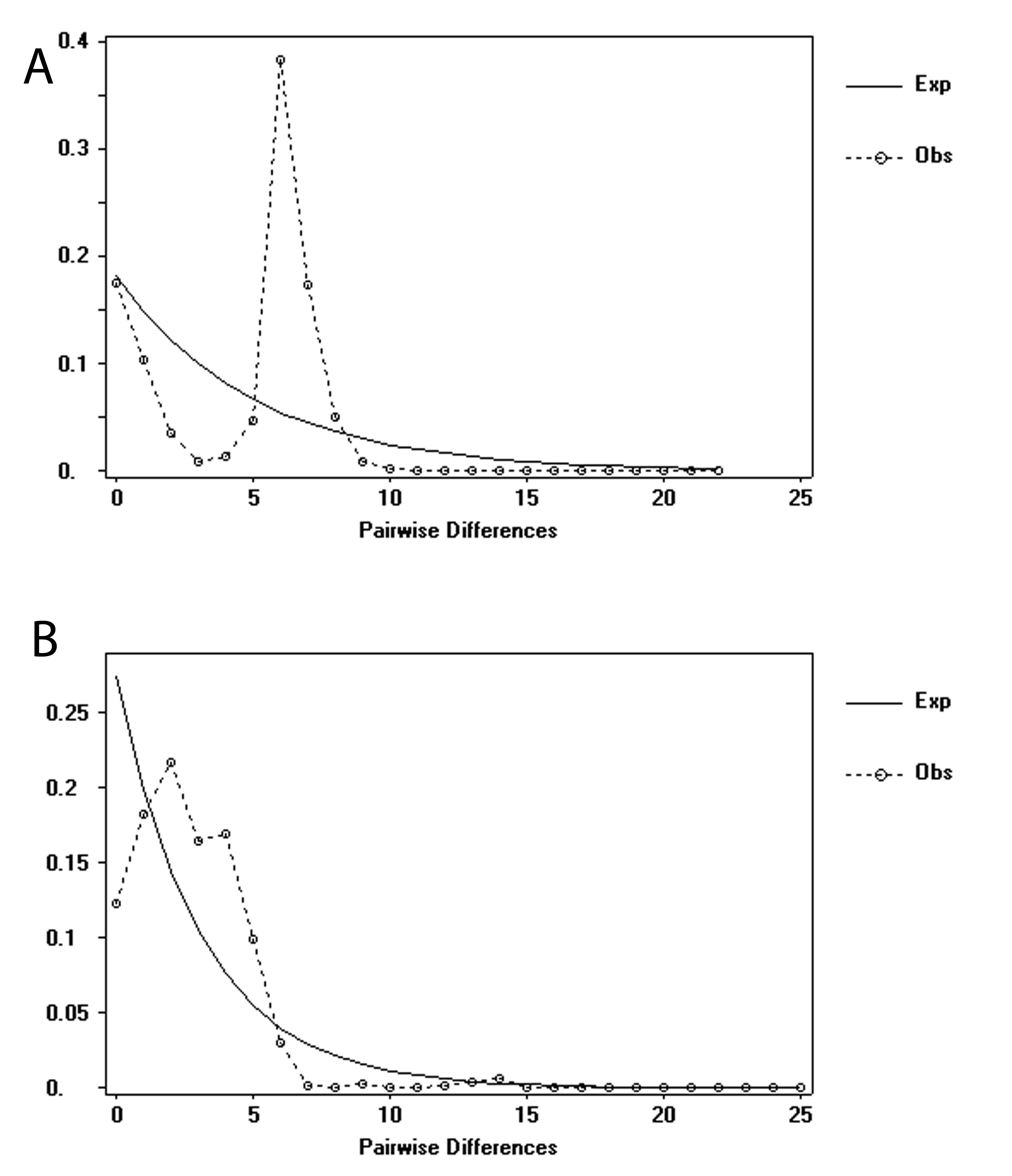

Supplement: Figure S2 — Mismatch distributions of pairwise nucleotide differences for total populations of S. mosellana using gene sequences of ND4 (A) and COX3 (B). Solid lines show the observed frequency distributions and dashed lines show the distribution expected under the sudden-expansion model. (TIF) [file pone.0078415.s002.tif]
