# Supplementary material for: Genetic Diversity and Population Structure of Sitodiplosis mosellana in Northern China
Source: PLoS One. 2013 Nov 12;8(11):e78415. doi: 10.1371/journal.pone.0078415 (PMC3827046; doi:10.1371/journal.pone.0078415)
Supplement: Table S1 — Pairwise genetic distances across all populations of S. mosellana based on mtDNA ND4 using Arlequin v3.5.1.2. (DOC) [file pone.0078415.s003.doc]

| Code | 1 | 2 | 3 | 4 | 5 | 6 | 7 | 8 | 9 | 10 | 11 | 12 | 13 | 14 | 15 | 16 |
| --- | --- | --- | --- | --- | --- | --- | --- | --- | --- | --- | --- | --- | --- | --- | --- | --- |
| 1.LY | 0.000 |  |  |  |  |  |  |  |  |  |  |  |  |  |  |  |
| 2.JN | -0.008 | 0.000 |  |  |  |  |  |  |  |  |  |  |  |  |  |  |
| 3.FN | -0.003 | -0.009 | 0.000 |  |  |  |  |  |  |  |  |  |  |  |  |  |
| 4.XT | 0.141^＊＊^ | 0.076^＊^ | 0.145^＊＊^ | 0.000 |  |  |  |  |  |  |  |  |  |  |  |  |
| 5.XS | 0.084^＊^ | 0.031 | 0.072^＊^ | -0.028 | 0.000 |  |  |  |  |  |  |  |  |  |  |  |
| 6.TJ | 0.088^＊^ | 0.038 | 0.068^＊^ | -0.014 | -0.039 | 0.000 |  |  |  |  |  |  |  |  |  |  |
| 7.BJ | -0.008 | -0.012 | -0.033 | 0.114^＊＊^ | 0.048 | 0.044 | 0.000 |  |  |  |  |  |  |  |  |  |
| 8.NY | 0.116^＊＊^ | 0.055^＊^ | 0.118^＊＊^ | -0.035 | -0.033 | -0.020 | 0.089^＊^ | 0.000 |  |  |  |  |  |  |  |  |
| 9.HX | 0.183^＊＊^ | 0.122^＊＊^ | 0.227^＊＊^ | 0.034 | 0.067^＊^ | 0.088^＊^ | 0.186^＊＊^ | 0.038 | 0.000 |  |  |  |  |  |  |  |
| 10.LC | 0.063^＊^ | 0.050^＊^ | 0.101^＊＊^ | 0.185^＊＊^ | 0.149^＊＊^ | 0.173^＊＊^ | 0.089^＊^ | 0.162^＊＊^ | 0.174^＊＊^ | 0.000 |  |  |  |  |  |  |
| 11.HuaX | 0.404^＊＊^ | 0.387^＊＊^ | 0.476^＊＊^ | 0.501^＊＊^ | 0.490^＊＊^ | 0.511^＊＊^ | 0.452^＊＊^ | 0.487^＊＊^ | 0.440^＊＊^ | 0.207^＊＊^ | 0.000 |  |  |  |  |  |
| 12.ZZ | 0.239^＊＊^ | 0.217^＊＊^ | 0.305^＊＊^ | 0.331^＊＊^ | 0.319^＊＊^ | 0.345^＊＊^ | 0.285^＊＊^ | 0.314^＊＊^ | 0.267^＊＊^ | 0.073^＊＊^ | 0.046^＊＊^ | 0.000 |  |  |  |  |
| 13.LF | 0.336^＊＊^ | 0.329^＊＊^ | 0.405^＊＊^ | 0.458^＊＊^ | 0.439^＊＊^ | 0.454^＊＊^ | 0.380^＊＊^ | 0.441^＊＊^ | 0.398^＊＊^ | 0.166^＊＊^ | 0.036 | 0.055^＊^ | 0.000 |  |  |  |
| 14.LT | 0.278^＊＊^ | 0.255^＊＊^ | 0.350^＊＊^ | 0.379^＊＊^ | 0.366^＊＊^ | 0.394^＊＊^ | 0.328^＊＊^ | 0.360^＊＊^ | 0.309^＊＊^ | 0.124^＊＊^ | 0.140^＊＊^ | 0.055^＊^ | 0.167^＊＊^ | 0.000 |  |  |
| 15.WW | 0.446^＊＊^ | 0.431^＊＊^ | 0.519^＊＊^ | 0.543^＊＊^ | 0.533^＊＊^ | 0.552^＊＊^ | 0.494^＊＊^ | 0.531^＊＊^ | 0.484^＊＊^ | 0.257^＊＊^ | 0.009 | 0.086^＊^ | 0.040 | 0.221^＊＊^ | 0.000 |  |
| 16.YC | 0.336^＊＊^ | 0.316^＊＊^ | 0.403^＊＊^ | 0.428^＊＊^ | 0.417^＊＊^ | 0.440^＊＊^ | 0.381^＊＊^ | 0.412^＊＊^ | 0.366^＊＊^ | 0.173^＊＊^ | 0.116^＊＊^ | 0.067^＊^ | 0.117^＊＊^ | 0.165^＊＊^ | 0.142^＊＊^ | 0.000 |

＊＊

Significance of comparisons indicated as follows: **P* < 0.05; ***P* < 0.001
