# Supplementary material for: Genetic Diversity and Population Structure of Sitodiplosis mosellana in Northern China
Source: PLoS One. 2013 Nov 12;8(11):e78415. doi: 10.1371/journal.pone.0078415 (PMC3827046; doi:10.1371/journal.pone.0078415)
Supplement: Table S2 — Analysis of molecular variance of S. mosellana based on mtDNA ND4 ( K = 2). (DOC) [file pone.0078415.s004.doc]

| Source of variation | d.f. | Percentage of variation | *P* value | Fixation indices |
| --- | --- | --- | --- | --- |
| Among groups | 1 | 50.14 | < 0.001 | *φ_CT_* = 0.501 |
| Among populations  within groups | 14 | 6.13 | < 0.001 | *φ_SC_* = 0.123 |
| Within populations | 304 | 43.73 | < 0.001 | *φ_ST_* = 0.563 |

Two groups of 16 *S. mosellana* populations were identified by pairwise *F*_ST_ and STRUCTURE (the eastern group: LY+JN+FN+XS+XT+TJ+BJ+NY+HX+LC; the western group: HuaX+ZZ+LF+LT+WW+YC).
