# Supplementary material for: Genetic Diversity and Population Structure of Sitodiplosis mosellana in Northern China
Source: PLoS One. 2013 Nov 12;8(11):e78415. doi: 10.1371/journal.pone.0078415 (PMC3827046; doi:10.1371/journal.pone.0078415)
Supplement: Table S3 — Analyses of natural tests of Tajima's D and Fu's Fs and bottlenecks in 16 populations of S. mosellana . (DOC) [file pone.0078415.s005.doc]

| Code | Natural test | | Wilcoxon sign rank test | | |
| --- | --- | --- | --- | --- | --- |
|  | Tajima’S *D* (*P*’s value) | Fu’s *F_S_* (*P*’s value) | I.A.M | T.P.M | S.M.M. |
| LY | 1.206 (0.908) | 1.614 (0.821) | 0.063 | 0.156 | 0.906 |
| JN | 0.443 (0.695) | -0.023 (0.538) | 0.438 | 0.438 | 0.438 |
| FN | 1.090 (0.883) | 1.974 (0.856) | 0.438 | 0.438 | 0.562 |
| XT | 0.721 (0.799) | 2.131 (0.860) | 0.063 | 0.438 | 0.563 |
| XS | 1.403 (0.929) | 3.519 (0.942) | 0.063 | 0.438 | 0.844 |
| TJ | 2.533 (0.992) | 5.275 (0.980) | 0.063 | 0.438 | 0.563 |
| BJ | 1.512 (0.944) | 1.392 (0.787) | 0.094 | 0.438 | 0.844 |
| NY | 1.027 (0.860) | 3.110 (0.928) | 0.438 | 0.563 | 0.906 |
| HX | -1.434 (0.067) | -1.156 (0.240) | 0.031 | 0.032 | 0.844 |
| LC | 0.844 (0.830) | 0.717 (0.661) | 0.062 | 0.563 | 0.938 |
| HuaX | -2.047 (0.006) | 0.357 (0.580) | 0.156 | 0.844 | 0.969 |
| ZZ | -0.519 (0.332) | -01.633 (0.217) | 0.032 | 0.065 | 0.438 |
| LF | 0.708 (0.780) | 4.627 (0.967) | 0.438 | 0.906 | 1.000 |
| LT | 0.985 (0.868) | 0.535 (0.568) | 0.563 | 0.906 | 0.969 |
| WW | -1.227 (0.113) | -1.751 (0.833) | 0.438 | 0.906 | 0.906 |
| NX | -1.349 (0.081) | -0.084 (0.461) | 0.063 | 0.156 | 0.563 |
| Eastern group | -0.524 (0.352) | -6.890 (0.041) | 0.063 | 0.438 | 0.844 |
| Western group | -1.373 (0.055) | -6.396 (0.017) | 0.031 | 0.969 | 1.000 |
| Total | -0.5303 (0.371) | -14.281 (0.004) | 0.063 | 0.438 | 0.563 |

*P* values for Tajima’s *D* and Fu’s *F_s_* indicate the probability that the simulated statistic will be more negative than the observed statistic.

Parameters for T.P.M: variance = 30.00, proportion of SMM in TPM = 70.00%; estimation based on 1,000 replications. Wilcoxon’s signed rank test *P* tests represent one-tailed probabilities for heterozygosity excess. I.A.M: infinite allele model; T.P.M: two-phase model; S.M.M: stepwise mutation model.
